# Multi-Output Machine Learning for Prediction of Postoperative Outcomes After Cardiac Surgery Using Patient Blood Management Biomarkers

**DOI:** 10.3390/jcm15114221

**Published:** 2026-05-29

**Authors:** Henrique Coelho, Diana Paupério, Fernando Silva, Maria Inês Barbosa, Pedro Ribeiro, Marta Correia, Pedro Miguel Rodrigues

**Affiliations:** 1 CBQF—Centro de Biotecnologia e Química Fina—Laboratório Associado, Escola Superior de Biotecnologia, Universidade Católica Portuguesa, 4169-005 Porto, Portugal; mibarbosa@ucp.pt (M.I.B.); s-pmsbribeiro@ucp.pt (P.R.); mmcorreia@ucp.pt (M.C.); pmrodrigues@ucp.pt (P.M.R.); 2 Serviço de Hematologia, Unidade Local de Saúde de Vila Nova de Gaia e Espinho, 4434-502 Vila Nova de Gaia, Portugal; 3 Departamento de Ciências Médicas, Universidade de Aveiro, 3810-193 Aveiro, Portugal; 70836@ulsra.min-saude.pt; 4 Serviço de Anestesiologia, Unidade Local de Saúde de Vila Nova de Gaia e Espinho, 4434-502 Vila Nova de Gaia, Portugal; diana.pauperio@ulsge.min-saude.pt; 5 Serviço de Hematologia, Unidade Local de Saúde da Região de Aveiro, 3814-501 Aveiro, Portugal

**Keywords:** cardiac surgery, machine learning, multi-output regression, patient blood management, postoperative complications

## Abstract

**Background/Objectives**: Postoperative complications following adult cardiac surgery are biologically interrelated, yet most machine learning models predict single outcomes. We developed an explainable multi-output model integrating routinely collected clinical variables and patient blood management (PBM) biomarkers to predict multiple postoperative outcomes simultaneously, with complementary mono-output analyses for selected endpoints. **Methods**: This retrospective single-center cohort included 1414 adults undergoing cardiac surgery. In total, 513 complete cases were analyzed. Thirteen outcomes were modeled, including major binary complications and ICU/ward length of stay. An initial 80:20 train–test split was used only for algorithm screening across six candidate multi-output regressors and training-set-defined feature subsets. The selected regressor was then evaluated across five random states, and global permutation feature importance was used for multi-output explainability. Mono-output binary analyses using the selected regressor and the same training-set-only feature-selection workflow were evaluated along with accuracy, precision, recall/sensitivity, and F1-scores. **Results**: The Decision Tree Regressor was selected. Across five random states, global multi-output performance was R^2^ = 0.83, MSE = 1.296, RMSE = 1.132, MAE = 0.298, and MAPE = 0.128. Based on global multi-output permutation importance, creatinine, ferritin, platelet count, estimated glomerular filtration rate, preoperative red blood cell units, and EuroSCORE II were ranked the highest. Atrial fibrillation had the lowest mono-output F1-score (0.719), whereas acute kidney injury, postoperative bleeding, infection, and 1-year hospital readmission yielded F1-scores of 0.928, 0.970, 0.963, and 0.975, respectively. **Conclusions**: This proof-of-concept study shows the feasibility of explainable multi-output modeling for postoperative outcomes after adult cardiac surgery using clinical and PBM variables. However, external validation is required prior to clinical use.

## 1. Introduction

Perioperative complications and adverse outcomes following adult cardiac surgery remain a major cause of morbidity, mortality, and healthcare utilization. Bleeding, transfusion, acute kidney injury (AKI), atrial fibrillation (AF), infection, respiratory complications, prolonged intensive care unit (ICU) stay, and late mortality often cluster, reflecting shared mechanisms such as inflammation, coagulation abnormalities, endothelial dysfunction, and impaired organ perfusion [1,2]. These domains overlap with established cardiac surgery risk and quality frameworks, including the Society of Thoracic Surgeons adult cardiac surgery risk models and the European System for Cardiac Operative Risk Evaluation (EuroSCORE II), which include renal, respiratory, infectious, neurological, mortality, functional status, and resource-utilization outcomes [3,4]. Machine learning (ML) approaches have improved the prediction of individual postoperative outcomes in cardiac surgery, including hemorrhage, venous thromboembolism, stroke, AKI, AF, gastrointestinal bleeding, and short-term mortality [5,6,7,8,9,10,11,12,13]. However, most studies rely on single-task models that treat each complication independently despite overlapping predictors and shared pathophysiology. Additional limitations include inconsistent predictor selection, potential bias and overfitting, and limited external validation; in real-world cardiovascular settings, ML models do not always outperform conventional risk scores [14,15].

Patient blood management (PBM) provides a clinically grounded framework for perioperative risk assessment in adult cardiac surgery. Contemporary PBM guidelines emphasize anemia management, coagulation and bleeding control, transfusion exposure, and hemostatic optimization [16]. Biomarkers such as hemoglobin, ferritin, folic acid, vitamin B_12_, platelet count, and coagulation indices reflect the erythropoietic reserve, inflammation status, and hemostatic balance. Although PBM variables can improve the prediction of bleeding and transfusion outcomes [17,18], most models primarily focus on hemoglobin and often overlook biomarkers related to iron metabolism and micronutrient deficiencies. Recent ML research in valve surgery has also identified ferritin, folic acid, and vitamin B_12_ as informative predictors of intraoperative red blood cell (RBC) transfusion, infection, bleeding, and prolonged hospitalization, supporting the inclusion of broader PBM biomarkers beyond hemoglobin alone [19,20].

Structured PBM pathways are particularly relevant in cardiac surgery because anemia, bleeding, transfusion exposure, renal dysfunction, and infection are closely interconnected and may affect both early and longer-term outcomes. A recently published Portuguese PBM implementation protocol highlights the practical role of preoperative anemia assessment, hemoglobin optimization, coagulation abnormality management, blood conservation strategies, viscoelastic-test-guided transfusion practice, and cell salvage [21]. In parallel, a recent systematic review of artificial intelligence in PBM showed applications in anemia detection, bleeding risk stratification, transfusion prediction, transfusion safety, and blood bank operations while emphasizing the need for external validation, explainability, workflow integration, and prospective evaluation [22].

Multi-output ML can model correlated postoperative outcomes within a single framework, as this task is related to multi-task- and tensor-based learning approaches that exploit structured predictor groups and correlations among multiple clinical outcomes [23,24,25]. Previous Bayesian network studies and registry analyses support joint postoperative prediction in cardiac surgery [26,27], and emerging studies also suggest that ML-derived physiological markers, such as artificial intelligence-based ECG-derived age, can capture overall patient vulnerability and predict multiple postoperative complications [28]. Recent healthcare risk-prediction studies further emphasize the value of visualization and interpretable outputs for clinically understandable risk stratification [29]. Nevertheless, explainable multi-output models that integrate PBM biomarkers remain uncommon in adult cardiac surgery.

We therefore developed and internally evaluated an explainable multi-output ML regression model integrating routinely collected preoperative clinical variables and PBM biomarkers in adults undergoing cardiac surgery. The model was designed to predict thirteen postoperative outcomes, comprising binary postoperative endpoints and length-of-stay outcomes, simultaneously while providing global feature-importance estimates. Complementary mono-output sensitivity analyses were performed for five clinically representative binary endpoints relevant to cardiac surgery and PBM, namely AF, AKI, postoperative bleeding, infection, and 1-year hospital readmission.

## 2. Materials and Methods

**Figure 1:**
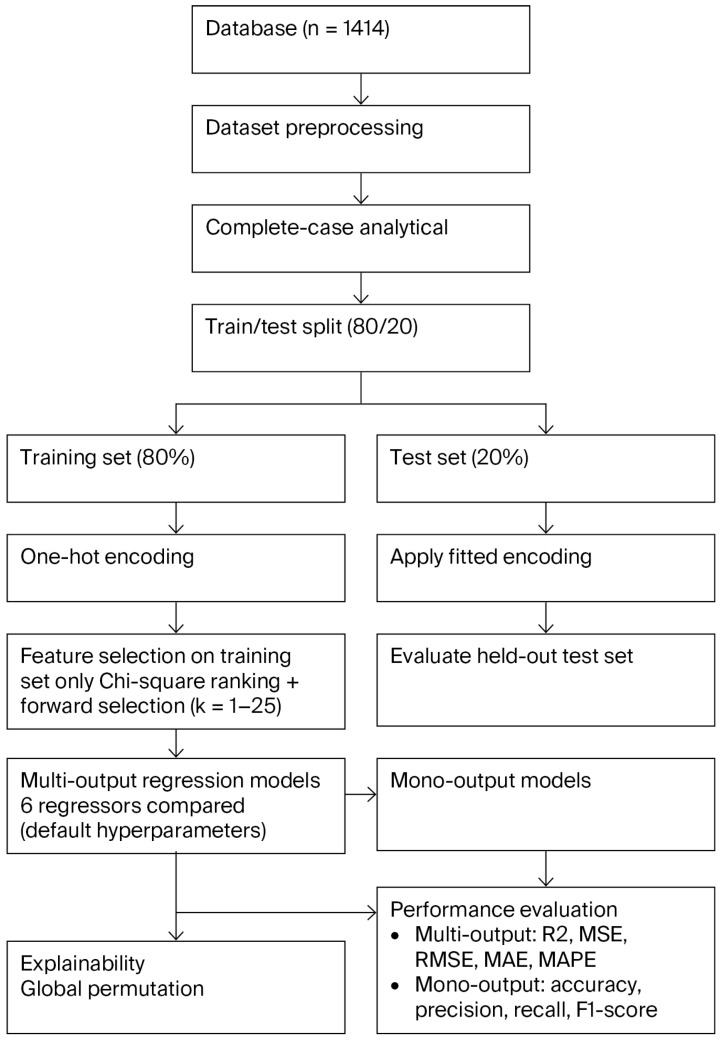
Workflow of the multi-output and mono-output machine learning pipeline. The complete-case cohort was split into training and held-out test sets across five random states. One-hot encoding and feature selection were fitted within the training set, and six multi-output regressors were screened; the selected regressor was applied to mono-output analyses, and global permutation feature importance was used for multi-output explainability.

All analyses were performed in Python 3.9.2 (Python Software Foundation, Wilmington, DE, USA) using scikit-learn Python library version 1.6.1, and the workflow comprised dataset preparation, preprocessing, feature selection, model training, and performance evaluation (Figure 1).

### 2.1. Study Population

This retrospective single-center dataset included 1414 consecutive adults undergoing cardiac surgery from January 2018 to September 2024, and surgical procedures were classified into several predefined categories: coronary artery bypass grafting (CABG), aortic valve surgery, mitral valve replacement (MVR), mitral valve repair, ascending aorta replacement (AAR), Bentall procedure, combined surgery, minimally invasive direct coronary artery bypass (MIDCAB), atrial septal defect (ASD) closure/repair, double-valve surgery, and triple-valve surgery.

Records with missing predictor or outcome data were removed, and the resulting complete-case analytical cohort comprised 513 patients. No imputation was performed in the primary analysis.

### 2.2. Outcomes

Thirteen postoperative outcomes were modeled: AF, myocardial infarction, stroke, acute respiratory distress syndrome (ARDS), infection, AKI, postoperative bleeding, postoperative in-hospital mortality, 1-year mortality, 1-year reintervention, 1-year hospital readmission, ICU length of stay, and ward length of stay.

The first eleven outcomes were binary endpoints, and ICU and ward length of stay were retained as continuous numerical outcomes on their original scale. Binary outcomes were encoded as 0/1 variables, with 0 indicating absence and 1 indicating event occurrence. This coding allowed binary postoperative endpoints and continuous length-of-stay outcomes to be included in a unified multi-output target matrix.

### 2.3. Predictors

Candidate predictors included demographic characteristics, comorbidities, surgical- and treatment-related variables, and routinely collected laboratory and PBM biomarkers.

Demographic and clinical variables included age, sex, body mass index, smoking status, alcohol use, hypertension, diabetes mellitus, chronic kidney disease, dyslipidemia, and chronic obstructive pulmonary disease.

Laboratory and PBM variables included hemoglobin measured at the preoperative PBM consultation and immediately before surgery, ferritin, platelet count, creatinine, the estimated glomerular filtration rate (eGFR), the international normalized ratio (INR), folic acid deficiency, and vitamin B_12_ deficiency.

Treatment-related variables included intravenous iron, erythropoietin, folic acid and vitamin B_12_ supplementation, antiplatelet therapy, anticoagulation, and preoperative RBC units.

Risk and surgical variables included New York Heart Association (NYHA) and American Society of Anesthesiologists (ASA) classes, EuroSCORE II, and surgery type. Surgery type was recorded as a multicategorical procedural variable using the predefined surgical categories described above, and variables labeled as target outcomes in the data dictionary were included in the multi-output target matrix and were not used as predictors.

Only variables available before surgery were eligible as predictors. Specifically, postoperative complications, outcome-defining variables, and downstream variables directly resulting from postoperative events were excluded from the predictor matrix. The variable preoperative RBC units referred exclusively to red blood cell units administered before the index operation and did not include intraoperative or postoperative transfusions. Information on variable definitions, coding, timing, and analytical role is provided in Supplementary Table S1.

### 2.4. Data Preprocessing and Partitioning

The modeling workflow comprised two consecutive stages: First, a single 80:20 train–test split was used for algorithm screening, comparing candidate regressors across progressively larger feature subsets defined within the training set. The best-performing regressor from this screening step was then carried forward. Second, after regressor selection, the complete modeling pipeline for the selected regressor was repeated across five fixed random states, with performance across these partitions used as the primary internal generalization estimate.

Within each partition, categorical variables, including surgery type and sex, were one-hot encoded (dummy coding) using the training data only [30], and the fitted encoding structure was subsequently applied to the corresponding held-out test set. For surgery type, this encoding generated binary indicators for the predefined procedural categories.

Except for the planned held-out evaluation used for regressor screening in the first stage, all preprocessing, feature-ranking/subset-definition, and model-fitting steps capable of learning data-derived transformations or model parameters were restricted to the training set for each split. In the algorithm-screening stage, the held-out partition was only used to evaluate trained candidate pipelines and select the regressor to carry forward. In contrast, the held-out partitions were only used to estimate the performance of the selected pipeline in the repeated-random-state evaluation, and no encoders, model parameters, decision thresholds, or hyperparameters were fitted or optimized using test-set labels.

No additional scaling or normalization was applied to the predictor variables, and no oversampling, undersampling, class-weighting strategy, or Synthetic Minority Oversampling Technique (SMOTE) was used in the primary multi-output regression framework. Event imbalance was documented by reporting outcome prevalence/distribution and, for binary endpoints with sufficient event counts, threshold-based endpoint-level classification metrics. Five fixed random seeds were used for repeated train–test partitioning, and estimator-level random-state parameters were fixed where supported to improve reproducibility.

### 2.5. Feature-Selection Strategy

Predictors were ranked using chi-square (χ^2^) scores as a univariate filter method. Since the target set included both binary endpoints and continuous length-of-stay outcomes, the χ^2^-based ranking was calculated using the binary endpoints only. For each predictor, χ^2^ scores were calculated separately for each binary endpoint and then averaged to generate a global ranking across the binary outcome set.

All predictors submitted to χ^2^ scoring were encoded as non-negative values. Categorical predictors were represented by one-hot encoding, while continuous predictors were retained on their original non-negative clinical scales. Additionally, continuous predictors were not discretized; therefore, χ^2^ ranking was used as an exploratory screening step rather than as a definitive measure of predictor importance. Feature selection was performed within the training set only.

Models were then trained with progressively larger cumulative subsets of ranked predictors, from *k* = 1 to *k* = 25, to examine performance across feature-set sizes. In the multi-output analysis, this feature-selection strategy was used for model development and algorithm comparison.

For the mono-output analyses, the same feature-selection strategy was applied separately to each binary endpoint using the corresponding training set only, and the predictor subset retained for each mono-output model was recorded, as shown in Supplementary Table S2. The retained variables are reported only as selected model inputs and should not be interpreted as measures of predictor importance or causal effects.

### 2.6. Model Architecture

A multi-output regression framework was used to predict all postoperative outcomes simultaneously by fitting one regression model per target variable.

Binary endpoints were encoded as 0/1 variables, whereas ICU and ward length-of-stay outcomes were retained as continuous numerical targets. This target structure enabled binary and continuous outcomes to be handled within a unified regression-based multi-output framework.

Six Scikit-learn regression algorithms [31] were compared within this framework (Table 1), and all algorithms were implemented using default scikit-learn hyperparameters. Random-state parameters were fixed when applicable. No grid-search, randomized-search, or cross-validation-based hyperparameter optimization was performed, and no hyperparameters were selected based on held-out test-set performance.

The regressor selected in the multi-output algorithm-screening step was subsequently used for all mono-output analyses. Each mono-output model followed the same preprocessing, train–test partitioning, training-set-only feature-selection, model-fitting, and held-out evaluation workflow used in the multi-output analysis, but it was fitted separately for each binary endpoint. Mono-output analyses were reported for five prespecified representative endpoints: AF, AKI, postoperative bleeding, infection, and 1-year hospital readmission. Global multi-output regression metrics and permutation-based explainability were not calculated for the mono-output models; instead, mono-output binary endpoints were evaluated with threshold-based classification metrics, and predictors retained by feature selection were reported for each endpoint.

### 2.7. Performance Evaluation

Performance on the held-out test set for each partition was assessed using four regression metrics: coefficient of determination (R^2^), mean squared error (MSE), root mean squared error (RMSE), and mean absolute error (MAE) [32]. Mean absolute percentage error (MAPE) was additionally reported as a secondary descriptive error metric. For the multi-output analysis, each metric was calculated for each outcome and then averaged across outputs to obtain a global estimate. During MAPE calculation, zero-valued observations were handled using numerical stabilization.

Formally, let *y_ij_* denote the true value for the *i*-th sample and *j*-th outcome, and let ${\bar{y}}_{\mathrm{ij}}$ be the corresponding predicted value. The dataset contains *n* samples and *M* output variables, where *i* = 1, …, *n* indexes samples and *j* = 1, …, *M* indexes outcomes. The mean observed value for each outcome is denoted by ${\bar{y}}_{j}$, with the global multi-output regression metrics defined as follows:

- Coefficient of Determination (R^2^): R^2^ represents the proportion of variance in the dependent variable predictable from the independent variables.

$$R^{2}=\left(\frac{1}{M}\right)\cdot \Sigma_{\left\{j=1:M\right\}}\left[1-\frac{\left(\Sigma_{\left\{i=1:n\right\}}{\left(y_{\left\{i,j\right\}}-{\hat{y}}_{\left\{i,j\right\}}\right)}^{2}\right)}{\left(\Sigma_{\left\{i=1:n\right\}}{\left(y_{\left\{i,j\right\}}-{\bar{y}}_{\left\{j\right\}}\right)}^{2}\right)}\right]$$

- Mean Squared Error (MSE): MSE measures the average of the squares of the errors.

$$MSE=\left(\frac{1}{M}\right)\cdot \Sigma_{\left\{j=1:M\right\}}\left[\left(\frac{1}{n}\right)\cdot \Sigma_{\left\{i=1:n\right\}}{\left(y_{\left\{i,j\right\}}-{\hat{y}}_{\left\{i,j\right\}}\right)}^{2}\right]$$

- Root Mean Squared Error (RMSE): RMSE is the square root of the MSE, providing error in the same units as the target variable.

$$RMSE=\left(\frac{1}{M}\right)\cdot \Sigma_{\left\{j=1:M\right\}}\left[\sqrt{\left(\frac{1}{n}\right)\cdot \Sigma_{\left\{i=1:n\right\}}{\left(y_{\left\{i,j\right\}}-{\hat{y}}_{\left\{i,j\right\}}\right)}^{2}}\right]$$

- Mean Absolute Error (MAE): MAE measures the average magnitude of the errors in a set of predictions, without considering their direction.

$$MAE=\left(\frac{1}{M}\right)\cdot \Sigma_{\left\{j=1:M\right\}}\left[\left(\frac{1}{n}\right)\cdot \Sigma_{\left\{i=1:n\right\}}\left|y_{\left\{i,j\right\}}-{\hat{y}}_{\left\{i,j\right\}}\right|\right]$$

- Mean Absolute Percentage Error (MAPE): MAPE measures the accuracy as a percentage of the error relative to the actual values.

$$MAPE=\left(\frac{1}{M}\right)\cdot \Sigma_{\left\{j=1:M\right\}}\left[\left(\frac{1}{n}\right)\cdot \Sigma_{\left\{i=1:n\right\}}\left|\frac{\left(y_{\left\{i,j\right\}}-{\hat{y}}_{\left\{i,j\right\}}\right)}{y_{\left\{i,j\right\}},\varepsilon }\right|\right]$$

To complement the global multi-output metrics, outcome-specific analyses were performed because the thirteen modeled outputs differed in scale, frequency, and clinical meaning.

For mono-output binary endpoints, continuous model outputs were thresholded at 0.5 to derive predicted class labels or endpoint-level classification metrics. Predicted labels were compared with the true 0/1 outcomes, and confusion-matrix-derived metrics were calculated according to standard definitions for binary classification performance [33]. The positive class corresponded to the occurrence of the postoperative endpoint. True positives (TPs), true negatives (TNs), false positives (FPs), and false negatives (FNs) were defined relative to this positive event class as follows:

Accuracy = (TP + TN)/(TP + TN + FP + FN)

Precision = TP/(TP + FP)

Recall/sensitivity = TP/(TP + FN)

F1-score = 2 × (Precision × Recall)/(Precision + Recall)

When a denominator was zero due to very low event counts, the corresponding metric was considered not estimable, and since several binary endpoints were rare or imbalanced, accuracy was interpreted together with precision, recall/sensitivity, and F1-scores.

For continuous outcomes, ICU and ward length of stay were retained on their original numerical scale and summarized as mean ± standard deviation in days. Classification metrics were not applicable to these continuous outcomes, and performance metrics were not estimated for ARDS and postoperative in-hospital mortality because each endpoint occurred in only one patient. The complete outcome-specific prevalence/distribution, predictors retained by mono-output feature selection, and mono-output performance results are reported in Supplementary Table S2.

### 2.8. Explainability Analysis

Permutation feature importance was used to estimate the global predictive importance of each predictor in the multi-output model [34], with importance estimated as the decrease in aggregated model performance after random permutation of each feature, thereby disrupting its relationship with the outcomes.

For each predictor, importance scores were summarized as mean values with standard deviations across repeated permutations, and because this analysis used an aggregated multi-output performance metric, importance scores were interpreted as global model-level estimates rather than outcome-specific or causal effects.

## 3. Results

### 3.1. Predictive Performance

**Figure 2:**
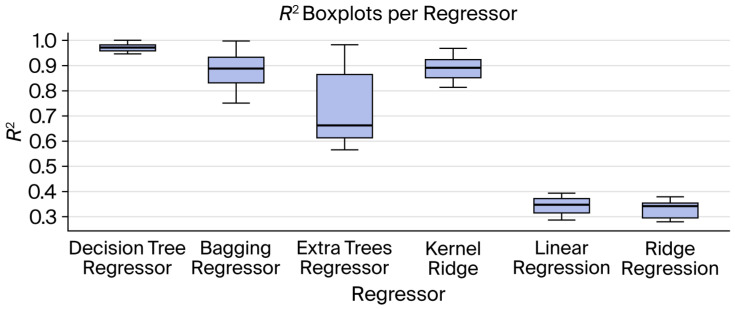
Predictive performance of six multi-output regression models across iterative feature-selection subsets in the initial algorithm-screening split. Boxplots show the distribution of R^2^ values for each regressor as progressively larger subsets of ranked predictors were used. Feature ranking and subset generation were performed within the training set only; the held-out test set was used to estimate performance after model fitting and to support regressor screening. Center lines indicate medians; boxes indicate interquartile ranges, and whiskers indicate the spread of values across feature-selection iterations.

In the initial algorithm-screening split, predictors were ranked using chi-square scores calculated from the training data only, and progressively larger cumulative subsets of these training-ranked predictors were then used to train each candidate multi-output regressor. The fitted candidate pipelines were evaluated on the corresponding held-out test partition, and the resulting performance distributions across feature-selection subsets are shown in Figure 2.

Tree-based models, particularly the Decision Tree, Extra Trees, and Bagging Regressors, yielded higher R^2^ distributions than linear models in the initial screening split. Kernel Ridge Regression also achieved high R^2^ values. Based on the screening results in Figure 2, the Decision Tree Regressor was selected for the subsequent multi-output and mono-output analyses.

After regressor selection, the full Decision Tree pipeline was repeated across five random states. This evaluation yielded the primary internal generalization estimates: R^2^ = 0.83, MSE = 1.296, RMSE = 1.132, MAE = 0.298, and MAPE = 0.128.

Each modeled output was then examined separately, with outcome-specific prevalence/distribution, predictors retained by mono-output feature selection, and mono-output predictive performance reported in Supplementary Table S2. Among binary endpoints, AF was the most frequent postoperative complication (146/513, 28.46%), followed by AKI (84/513, 16.37%) and 1-year hospital readmission (56/513, 10.92%). Less frequent endpoints included postoperative bleeding and 1-year reintervention (27/513 each, 5.26%), 1-year mortality (22/513, 4.29%), myocardial infarction (10/513, 1.95%), and stroke (6/513, 1.17%). ARDS and postoperative in-hospital mortality each occurred in only one patient (0.19%); therefore, mono-output performance metrics were not estimated for these two endpoints. ICU and ward length of stay were 2.71 ± 3.35 and 3.99 ± 3.17 days, respectively.

Table 2 reports mono-output classification metrics and the predictors retained by endpoint-specific feature selection for the five prespecified representative binary endpoints. AF had the lowest mono-output performance among these endpoints (F1-score = 0.719), and AKI, postoperative bleeding, infection, and 1-year hospital readmission had F1-scores of 0.928, 0.970, 0.963, and 0.975, respectively.

### 3.2. Contributions of Predictors and Explainability Analysis

**Figure 3:**
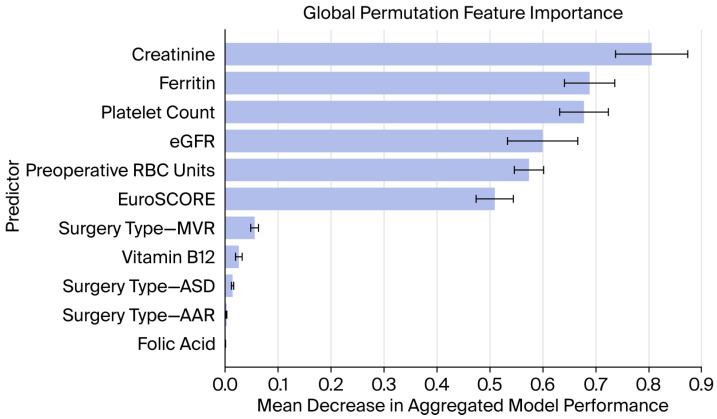
Global permutation feature-importance analysis of the multi-output regression model. Feature importance was estimated as the mean decrease in aggregated multi-output model performance after random permutation of each predictor variable. Larger values indicate greater loss of model performance after permutation, corresponding to greater global model-level importance. Error bars represent the standard deviation of the permutation importance scores across repeated permutations. MVR: mitral valve replacement; ASD: atrial septal defect; AAR: ascending aorta replacement; eGFR: estimated glomerular filtration rate; RBC: red blood cell.

Predictor importance in the final multi-output model is summarized in Table 3 and illustrated in Figure 3.

Creatinine showed the highest global permutation importance in the multi-output model, with a mean decrease in aggregated model performance (0.8066 ± 0.0685), followed by ferritin, platelet count, eGFR, preoperative RBC units, and EuroSCORE II. Preoperative RBC units were recorded before the index operation and did not include intraoperative or postoperative transfusion, and vitamin B_12_ deficiency and folic acid deficiency had lower mean importance scores (0.0263 and 0.0013, respectively).

## 4. Discussion

This study demonstrates the feasibility of an explainable multi-output ML framework for the simultaneous prediction of clinically related postoperative outcomes after adult cardiac surgery. Its main contribution is the integration of multiple postoperative outcomes within one biologically grounded and interpretable model rather than the isolated prediction of a single endpoint. By combining routinely collected clinical variables with PBM biomarkers, the proposed framework captures shared physiological vulnerability across postoperative complications and resource-utilization outcomes.

### 4.1. Positioning Within the Existing Literature

Most ML models in cardiac surgery target single outcomes such as bleeding, AKI, AF, or mortality [5,6,7,8,9,10,11,12,13]. More recent research on cardiac surgery has started to explore multi-task or multi-complication prediction using advanced representation-learning frameworks, indicating a shift beyond isolated single-endpoint models [35].

However, many models are still developed independently despite substantial overlap in risk factors and pathophysiological mechanisms across postoperative complications. Methodological concerns also remain common, including heterogeneous model development, limited external validation, potential overfitting, and possible overestimation of internal performance [14,22]. These concerns are consistent with recent PBM-focused evidence, in which AI models frequently achieved strong retrospective performance but were limited by heterogeneous reporting, imbalanced datasets, inconsistent validation strategies, limited prospective evaluation, and scarce workflow integration [22]. The present study should therefore be viewed as a proof-of-concept contribution within this evolving PBM-AI literature rather than as a clinically deployable prediction tool.

The multi-output approach used here addresses this gap by jointly modeling related postoperative outcomes. This strategy is consistent with previous Bayesian network- and registry-based studies supporting joint outcome prediction in cardiac surgery [26,27] while extending the concept through an explainable regression-based framework incorporating PBM-related predictors. The mono-output analyses complemented the global multi-output evaluation by describing endpoint-specific performance for AF, AKI, postoperative bleeding, infection, and 1-year hospital readmission.

### 4.2. Interpretation of Model Performance

The initial single train–test split was used as an algorithm-screening step to select the best-performing regressor across training-set-defined feature subsets. Because this split contributed to regressor selection, the repeated-random-state evaluation was used as the primary internal estimate of generalization. This distinction is important because single splits resulting in small- or moderate-sized medical datasets may be overly optimistic and sensitive to the specific random partition [36].

After selecting the Decision Tree Regressor, the full modeling pipeline showed strong internal performance across five random states. These results should be interpreted as proof-of-concept performance rather than as evidence of clinical readiness [37].

Global multi-output metrics provide summary descriptors of overall performance, but they may obscure heterogeneous performance across individual outcomes, especially rare or imbalanced binary endpoints. Because binary endpoints were encoded as 0/1 variables within a regression framework, continuous model outputs for these endpoints should be regarded as risk scores rather than calibrated probabilities. Therefore, endpoint-level evaluation remains essential when assessing potential clinical relevance [38,39,40].

The mono-output analyses showed endpoint-specific heterogeneity. AF had the lowest performance, whereas AKI, postoperative bleeding, infection, and 1-year hospital readmission showed higher internal performance. These analyses were designed to complement the global multi-output metrics and to describe model behavior across major postoperative domains in cardiac surgery and PBM.

Within this context, the findings support the feasibility of unified multi-output modeling rather than definitive clinical accuracy. The stronger performance of tree-based models is consistent with their ability to capture nonlinear effects and higher-order interactions in perioperative data. However, external validation in independent multicenter cohorts, together with calibration, threshold-independent metrics, and more complete outcome-specific evaluation, will be necessary before clinical use [38,39,40].

### 4.3. Biological Plausibility and the Role of PBM Biomarkers

A major strength of the proposed model is the biological plausibility of the predictors identified through global permutation feature importance analysis. Renal function and hematologic variables—especially creatinine, ferritin, platelet count, eGFR, and preoperative RBC units—made the largest contributions to aggregated multi-output model performance.

This pattern is clinically plausible. Renal dysfunction, impaired oxygen delivery, anemia-related vulnerability, altered hemostatic reserve, inflammation, and transfusion-related risk are closely interconnected in cardiac surgery [1,2,16], and prior studies have shown the importance of renal function, hematologic reserve, and transfusion-related variables for postoperative outcomes such as AKI, bleeding, and prolonged hospitalization [6,7,8,17,18,19]. In the present model, the prominence of renal and hematologic predictors suggests that the multi-output framework may be capturing a broader systemic vulnerability phenotype rather than isolated risk pathways for individual complications.

PBM-related biomarkers, including ferritin, folic acid, and vitamin B_12_, may add clinically relevant information beyond hemoglobin alone [41,42]. Ferritin reflects iron availability but may also be influenced by inflammation, while folic acid and vitamin B_12_ are central to erythropoiesis and may reflect broader nutritional and metabolic status [43,44,45]. Recent postoperative infection models in cardiac surgery have identified perioperative transfusion- and inflammation-related variables as important predictors, supporting links between PBM-related physiology and infectious morbidity [46]. Although micronutrient biomarkers contributed less strongly than renal and hematologic variables in the global permutation analysis, their inclusion supports the value of broader PBM profiling in perioperative risk stratification.

Clinically, PBM biomarkers may help identify modifiable vulnerability domains before surgery, including anemia, iron deficiency, impaired erythropoietic reserve, inflammation, renal dysfunction, and altered hemostatic capacity, and integrating these indicators into risk models could support targeted perioperative optimization, such as anemia or iron deficiency correction, review of antithrombotic therapy, transfusion planning, and closer perioperative surveillance.

### 4.4. Explainability and Clinical Credibility

Interpretability is essential for the clinical adoption of ML models. In this study, explainability was addressed with permutation feature importance, which provided a transparent ranking of variables contributing to model performance. However, permutation-based importance is model-dependent and should not be interpreted as evidence of causality [34].

Because importance scores were aggregated across all modeled outcomes, they should not be interpreted as predictor effects for specific postoperative complications. The mono-output sensitivity analyses improved endpoint-level performance reporting but did not provide outcome-specific permutation importance. Therefore, the feature-importance results should be considered global model-level explanations rather than local or complication-specific explanations.

Although outcome-specific SHAP or permutation-importance analyses were not performed, mono-output feature selection provided an endpoint-level summary of the predictors retained in each binary mono-output model. These variables should be interpreted as selected model inputs rather than as causal determinants, SHAP values, permutation feature importance, or ranked outcome-specific predictor effects.

The five mono-output endpoints summarized in Table 2 represent clinically relevant postoperative domains in adult cardiac surgery and PBM. In the present cohort, AF was the most frequent postoperative complication (146/513, 28.46%), consistent with studies describing postoperative AF as one of the most common complications after cardiac surgery, with reported incidences commonly ranging from approximately 15% to 40% depending on the monitoring strategy, definition, and procedure type [47]. AKI occurred in 84/513 patients (16.37%). This is within the broad range reported for cardiac surgery-associated AKI, which may reach approximately 30% depending on the definition and procedure type [48]. Infection occurred in 39/513 patients (7.60%), in line with a recent adult cardiac-surgery cohort with cardiopulmonary bypass that reported postoperative infection in 8.21% of patients [49]. Postoperative bleeding occurred in 27/513 patients (5.26%), a frequency compatible with clinically significant bleeding or re-exploration-related bleeding rates reported after cardiac surgery, although comparisons depend strongly on the bleeding definition used [50]. Finally, 1-year hospital readmission occurred in 56/513 patients (10.92%). Importantly, direct comparison with the literature is difficult because readmission estimates vary by procedure type, follow-up window, and whether readmission is all-cause or cause-specific; nevertheless, readmission is widely recognized as an important quality-of-care and resource-utilization outcome after cardiac surgery [51,52].

The predictors retained by mono-output feature selection were consistent with these clinical domains. AF retained NYHA class, BMI (body mass index), hypertension, chronic kidney disease, and COPD (chronic obstructive pulmonary disease), reflecting cardiopulmonary comorbidity and functional-status burden. AKI retained BMI, while creatinine and eGFR were among the most important global multi-output predictors, suggesting complementary clinical and renal-function signals. Postoperative bleeding retained the broadest predictor set, including preoperative hemoglobin, preoperative RBC units, ASA class, BMI, hypertension, diabetes mellitus, chronic kidney disease, dyslipidemia, and COPD, aligning with PBM domains related to anemia, transfusion exposure, comorbidity burden, and hemostatic vulnerability. Infection retained age, which may represent a general marker of physiological vulnerability, and 1-year hospital readmission retained diabetes mellitus, supporting the role of multimorbidity in downstream resource utilization.

The prominence of established clinical risk factors, particularly renal and hematologic markers, supports the biological plausibility of the model and reduces concern that predictions were mainly driven by spurious associations. Alignment between model behavior and known physiology may improve clinician confidence in future applications. Therefore, future studies should complement global permutation importance with outcome-specific interpretability, SHAP values, partial dependence plots, and feature-importance stability analyses.

### 4.5. Limitations and Future Directions

This study has several limitations. First, its retrospective single-center design may limit generalizability, as institutional practices, patient populations, PBM protocols, transfusion thresholds, surgical case mix, and outcome definitions may differ across centers. External multicenter validation is therefore required before the model can be considered for broader clinical use [37,39].

Second, the analysis used a complete-case cohort of 513 patients from an initial dataset of 1414 adults. Complete-case analysis reduced the available sample size and may have introduced selection bias if the patients with complete PBM biomarker data differed systematically from those excluded because of missingness. No imputation was performed in the primary analysis to avoid adding assumptions to a dataset with substantial biomarker missingness, but future studies should evaluate robust missing-data strategies and compare complete-case and imputed analyses [53].

Third, no learning-curve analysis, nested cross-validation, or hyperparameter optimization was performed. Default hyperparameters were used to provide a transparent baseline comparison across algorithm families, but this strategy limits the strength of model comparison and does not represent optimized model performance. Future work should optimize hyperparameters exclusively within the training data, preferably using nested cross-validation or independent validation cohorts, and assess learning curves to characterize sample-size effects and overfitting [36,37,39].

Fourth, several endpoints were rare, including ARDS, postoperative in-hospital mortality, stroke, myocardial infarction, 1-year mortality, and 1-year reintervention. Rare outcomes limit the stability and clinical interpretability of endpoint-specific performance metrics. Accordingly, mono-output analyses were only emphasized for selected representative endpoints with sufficient event counts and should be interpreted as exploratory internal sensitivity analyses [38,54].

Fifth, the outcome set was heterogeneous, including binary postoperative endpoints and continuous length-of-stay outcomes. The multi-output regression framework was useful as a proof-of-concept strategy, but future studies should compare this approach with dedicated classification models, mixed-type multi-task architectures, calibration methods, and threshold-independent metrics such as AUROC and AUPRC [35,38,39,40].

Sixth, chi-square-based feature ranking was used as an exploratory univariate screening method. This approach may not fully capture nonlinear associations, predictor interactions, or outcome-specific relevance, which requires careful handling of continuous predictors. Future analyses should compare this strategy with mutual information, recursive feature elimination, embedded model-based selection, and stability-based feature-selection methods [55,56].

Seventh, permutation feature importance provided useful model-level interpretability, but it did not provide patient-level explanations, causal insight, or outcome-specific importance estimates. Although Supplementary Table S2 reports predictors retained by mono-output feature selection, these selected predictors are not equivalent to outcome-specific SHAP values, permutation feature importance, or causal effects. Therefore, future work should include local interpretability methods, such as SHAP values, as well as outcome-specific feature-importance analyses [57].

Eighth, laboratory biomarkers such as ferritin, folate, and vitamin B_12_ may be influenced by dietary intake, physiological status, inflammation, assay methods, and biomarker cut-offs, potentially increasing heterogeneity across populations [42,43,44,45].

Ninth, standard tabular multi-output regression algorithms were used. However, the prediction task is conceptually related to tensor regression because predictors can be organized into structured feature groups, including patient characteristics, surgical variables, laboratory markers, and PBM indicators, while the outputs consist of multiple related postoperative outcomes. Tensor-based approaches may provide a more expressive framework for modeling low-rank feature–outcome structure, higher-order interactions, and correlations among postoperative outcomes. They may also be useful in future multicenter studies, where additional structure may arise from centers, surgical subgroups, or perioperative time points [23,24,25].

Tenth, the current framework does not include temporal or longitudinal physiological data. Future work incorporating time-dependent perioperative trajectories may improve predictive performance and enable more actionable perioperative decision support, as prior CABG readmission models have shown added value from longitudinal perioperative variables [58].

Finally, this study did not evaluate clinical implementation, decision curve analysis, prospective workflow integration, or cost-effectiveness. Before clinical deployment, the framework should be tested prospectively, integrated with electronic health records, evaluated for calibration and clinical utility, and assessed for its impact on PBM interventions, resource utilization, and patient outcomes [59,60].

## 5. Conclusions

In this retrospective proof-of-concept study, an explainable multi-output ML framework integrating routine clinical and PBM variables enabled the joint modeling of thirteen postoperative outcomes after adult cardiac surgery. An initial 80:20 train–test split was used for algorithm screening and regressor selection, and repeated evaluation across five random states provided the primary internal estimate of generalization.

Tree-based models showed the strongest internal performance, and renal-, hematologic-, and PBM-related markers were the main global contributors to the multi-output model, supporting the biological plausibility of the approach. Selected mono-output analyses for AF, AKI, postoperative bleeding, infection, and 1-year hospital readmission provided additional endpoint-level insight while remaining exploratory and internally validated only.

Overall, the findings support simultaneous prediction of correlated postoperative outcomes as a biologically coherent strategy for perioperative risk modeling. Before clinical implementation, the framework requires external multicenter validation, optimized model development with appropriate internal validation, calibration and clinical-utility assessment, fuller outcome-specific evaluation, and testing in larger datasets with more complete and longitudinal perioperative information.

## Figures and Tables

**Table 1:** Regression algorithms and hyperparameter settings used in the multi-output machine learning framework.

Regressor (Scikit-Learn Class)	Hyperparameters
Linear Regression (LinearRegression)	fit_intercept = True, copy_X = True
Ridge Regression (Ridge)	alpha = 1.0, fit_intercept = True, solver = “auto”
Decision Tree (DecisionTreeRegressor)	criterion = “squared_error”, splitter = “best”
Extra Trees Regressor (ExtraTreesRegressor)	n_estimators = 100, criterion = “squared_error”, random_state = 42
Bagging Regressor (BaggingRegressor)	n_estimators = 10, bootstrap = True, random_state = 17
Kernel Ridge (KernelRidge)	alpha = 1.0, kernel = “linear”, degree = 3

**Table 2:** Mono-output sensitivity analysis for selected binary postoperative endpoints. Accuracy, precision, recall/sensitivity, and F1-scores were calculated after thresholding model outputs to derive binary.

Outcome	Selected Predictors Retained by Mono-Output Feature Selection	Accuracy	Precision	Recall/ Sensitivity	F1-Score
Atrial fibrillation	*n* = 5: NYHA class; BMI; hypertension; chronic kidney disease; COPD	0.602	0.730	0.708	0.719
Acute kidney injury	*n* = 1: BMI	0.866	0.923	0.933	0.928
Postoperative bleeding	*n* = 9: preoperative hemoglobin; preoperative RBC units; ASA class; BMI; hypertension; diabetes mellitus; chronic kidney disease; dyslipidemia; COPD	0.944	0.970	0.971	0.970
Infection	*n* = 1: age	0.928	0.932	0.996	0.963
1-year hospital readmission	*n* = 1: diabetes mellitus	0.951	0.951	1.000	0.975

Note: ASA, American Society of Anesthesiologists; BMI, body mass index; COPD, chronic obstructive pulmonary disease; NYHA, New York Heart Association; RBC, red blood cell. Preoperative RBC units refer to units administered before the index operation and do not include intraoperative or postoperative transfusion.

**Table 3:** Permutation feature-importance scores for the multi-output regression model. Feature importance was estimated by measuring the decrease in aggregated model performance after randomly permutating each predictor variable. Higher values indicate greater influence on model performance.

Feature	Mean	Standard Deviation
Creatinine	0.8066	0.0685
Ferritin	0.6892	0.0478
Platelet Count	0.6785	0.0463
eGFR	0.6004	0.0667
Preoperative RBC Units	0.5745	0.0278
EuroSCORE II	0.5099	0.0350
Surgery Type—MVR	0.0561	0.0072
Vitamin B_12_ deficiency	0.0263	0.0061
Surgery Type—ASD	0.0144	0.0022
Surgery Type—AAR	0.0033	0.0005
Folic acid deficiency	0.0013	0.0001

Note: MVR: mitral valve replacement; ASD: atrial septal defect; AAR: ascending aorta replacement; eGFR: estimated glomerular filtration rate; RBC: red blood cell.

## Data Availability

The data presented in this study are available upon reasonable request from the corresponding author due to privacy and ethical restrictions. The analysis code used for preprocessing, feature selection, model fitting, performance evaluation, and generation of the reported results is publicly available at https://github.com/PeBapRi/multioutput-cardiac-surgery.git (accessed on 15 May 2026). The public repository does not contain patient-level data.

## Supplementary Materials

The following supporting information can be downloaded at https://www.mdpi.com/article/10.3390/jcm15114221/s1, Table S1: Data extraction template and coding scheme for the analytical dataset; Supplementary Table S2: Outcome-specific prevalence/distribution, predictors retained by mono-output feature selection, and mono-output predictive performance across the thirteen modeled outputs.

## Abbreviations

The following abbreviations are used in this manuscript:

AAR	Ascending aorta replacement
AF	Atrial fibrillation
AKI	Acute kidney injury
ARDS	Acute respiratory distress syndrome
ASA	American Society of Anesthesiologists
ASD	Atrial septal defect
AUPRC	Area under the precision–recall curve
AUROC	Area under the receiver operating characteristic curve
CABG	Coronary artery bypass grafting
eGFR	Estimated glomerular filtration rate
FNs	False negatives
FPs	False positives
ICU	Intensive care unit
INR	International normalized ratio
MAE	Mean absolute error
MAPE	Mean absolute percentage error
MIDCAB	Minimally invasive direct coronary artery bypass
ML	Machine learning
MSE	Mean squared error
MVR	Mitral valve replacement
NYHA	New York Heart Association
PBM	Patient blood management
R^2^	Coefficient of determination
RBC	Red blood cell
RMSE	Root mean squared error
SHAP	SHapley Additive exPlanations
SMOTE	Synthetic Minority Oversampling Technique
TNs	True negatives
TPs	True positives
