# Supplementary material for: Multi-Output Machine Learning for Prediction of Postoperative Outcomes After Cardiac Surgery Using Patient Blood Management Biomarkers
Source: J Clin Med. 2026 May 29;15(11):4221. doi: 10.3390/jcm15114221 (PMC13258426; doi:10.3390/jcm15114221)
Supplement: Supplementary file 1 [file jcm-15-04221-s001.zip › jcm-4307645-supplementary.pdf]

Supplementary Table S1. Data-extraction template and coding scheme for the analytical dataset. The table summarizes the variables collected, coding scheme, timing/source of collection, and use in the primary analysis. Variables labeled as target outcomes were included in the multi-output target matrix and were not used as predictors. Only variables labeled as predictors were eligible for model input. Intraoperative variables were collected for descriptive purposes but were not included in the primary preoperative prediction model. Binary outcomes were coded as 0 = no event and 1 = event. Binary treatments and comorbidities were coded as 0 = absent/not performed and 1 = present/performed. The table is provided as a variable dictionary and does not contain patient-level raw data.

Domain	Variable	Role in primary analysis	Definition / coding / unit	Timing/source	Notes
Identifier	Patient identifier	Identifier; removed before analysis	Internal administrative code only	Administrative database	Removed before model development
Outcome	AF	Target outcome	0 = no event; 1 = event	Postoperative record	Binary endpoint
Outcome	Myocardial infarction	Target outcome	0 = no event; 1 = event	Postoperative record	Binary endpoint
Outcome	ARDS	Target outcome	0 = no event; 1 = event	Postoperative record	Binary endpoint
Outcome	Stroke	Target outcome	0 = no event; 1 = event	Postoperative record	Binary endpoint
Outcome	Infection	Target outcome	0 = no event; 1 = event	Postoperative record	Binary endpoint
Outcome	AKI	Target outcome	0 = no event; 1 = event	Postoperative record	Binary endpoint
Outcome	Postoperative bleeding	Target outcome	0 = no event; 1 = event	Postoperative record	Binary endpoint
Outcome	Postoperative in-hospital mortality	Target outcome	Death occurring after the index cardiac surgery and before discharge from the index hospitalization; 0 = no; 1 = yes	Hospital record	Binary endpoint
Outcome	1-year mortality	Target outcome	All-cause death within 365 days after the index cardiac surgery; 0 = no; 1 = yes	Follow-up record	Binary endpoint
Outcome	1-year reintervention	Target outcome	0 = no event; 1 = event	Follow-up record	Binary endpoint
Outcome	1-year hospital readmission	Target outcome	0 = no event; 1 = event	Follow-up record	Binary endpoint
Outcome	ICU length of stay	Target outcome	Days	ICU record	Continuous outcome

Domain	Variable	Role in primary analysis	Definition / coding / unit	Timing/source	Notes
Outcome	Ward length of stay	Target outcome	Days	Hospital record	Continuous outcome
Demographic	Age	Preoperative predictor	Years	Preoperative record	Continuous predictor
Demographic	Sex	Preoperative predictor	0 = female; 1 = male	Preoperative record	Binary predictor
Surgical	Surgery type	Surgical predictor	Multicategorical procedural variable encoded as binary indicators for predefined surgical categories	Surgical record	Categories included CABG, aortic valve surgery, mitral valve replacement, Bentall procedure, combined surgery, MIDCAB, atrial septal defect closure/repair, double-valve surgery, mitral valve repair, and triple-valve surgery
Clinical	NYHA class	Preoperative predictor	0 = NYHA I-II; 1 = NYHA≥ III	Preoperative record	Binary-coded risk variable
Clinical	ASA class	Preoperative predictor	0 = ASA I–II; 1 = ASA ≥ III	Preoperative record	Binary-coded risk variable
Clinical	EuroSCORE II	Preoperative predictor	Operative mortality risk in percentage points (%)	Preoperative record	Continuous predictor risk variable
Clinical	BMI	Preoperative predictor	kg/m^2	Preoperative record	Continuous predictor
Comorbidity	Hypertension	Preoperative predictor	0 = absent; 1 = present	Preoperative record	Binary predictor
Comorbidity	Diabetes mellitus	Preoperative predictor	0 = absent; 1 = present	Preoperative record	Binary predictor
Comorbidity	Chronic kidney disease	Preoperative predictor	0 = absent; 1 = present	Preoperative record	Binary predictor
Comorbidity	Dyslipidemia	Preoperative predictor	0 = absent; 1 = present	Preoperative record	Binary predictor
Comorbidity	Chronic obstructive pulmonary disease	Preoperative predictor	0 = absent; 1 = present	Preoperative record	Binary predictor
Lifestyle	Smoking status	Preoperative predictor	0 = no; 1 = yes	Preoperative record	Binary predictor
Lifestyle	Alcohol use	Preoperative predictor	0 = no; 1 = yes	Preoperative record	Binary predictor
Laboratory/PBM	Hemoglobin at PBM consultation	Preoperative predictor	g/dL	Preoperative PBM consultation	Continuous predictor

Domain	Variable	Role in primary analysis	Definition / coding / unit	Timing/source	Notes
Laboratory/PBM	Immediate preoperative hemoglobin	Preoperative predictor	g/dL	Preoperative laboratory record	Continuous predictor
Laboratory/PBM	Ferritin	Preoperative predictor	ng/mL	Preoperative laboratory record	Continuous predictor
Laboratory/PBM	Folic acid deficiency	Preoperative predictor	0 = no deficiency; 1 = deficiency	Preoperative laboratory record	Binary predictor
Laboratory/PBM	Vitamin B12 deficiency	Preoperative predictor	0 = no deficiency; 1 = deficiency	Preoperative laboratory record	Binary predictor
Laboratory/PBM	Platelet count	Preoperative predictor	$\times 10^9$/L	Preoperative laboratory record	Continuous predictor
Laboratory/PBM	Creatinine	Preoperative predictor	mg/dL	Preoperative laboratory record	Continuous predictor
Laboratory/PBM	INR	Preoperative predictor	Numerical value	Preoperative laboratory record	Continuous predictor
Laboratory/PBM	eGFR	Preoperative predictor	mL/min/1.73 m^2	Preoperative laboratory record	Continuous predictor
Treatment	Antiplatelet therapy	Preoperative predictor	0 = no; 1 = yes	Preoperative medication record	Binary predictor
Treatment	Anticoagulation	Preoperative predictor	0 = no; 1 = yes	Preoperative medication record	Binary predictor
Treatment	Intravenous iron	Preoperative predictor	0 = not performed; 1 = performed	Preoperative PBM record	Binary predictor
Treatment	Folic acid supplementation	Preoperative predictor	0 = not performed; 1 = performed	Preoperative PBM record	Binary predictor
Treatment	Vitamin B12 supplementation	Preoperative predictor	0 = not performed; 1 = performed	Preoperative PBM record	Binary predictor
Treatment	Erythropoietin	Preoperative predictor	0 = not performed; 1 = performed	Preoperative PBM record	Binary predictor
Treatment	Preoperative RBC units	Preoperative predictor	Number of RBC units administered before the index operation	Preoperative record	Does not include intraoperative or postoperative transfusion

Domain	Variable	Role in primary analysis	Definition / coding / unit	Timing/source	Notes
Intraoperative	Tranexamic acid during surgery	Collected but not used in primary model	0 = no; 1 = yes	Intraoperative record	Excluded from the primary preoperative prediction model

Note: AF, atrial fibrillation; AKI, acute kidney injury; ARDS, acute respiratory distress syndrome; ASA, American Society of Anesthesiologists; BMI, body mass index; CABG, coronary artery bypass grafting; eGFR, estimated glomerular filtration rate; ICU, intensive care unit; INR, international normalized ratio; MIDCAB, minimally invasive direct coronary artery bypass; NYHA, New York Heart Association; PBM, patient blood management; RBC, red blood cell.

Supplementary Table S2. Outcome-specific prevalence/distribution, predictors retained by mono-output feature selection, and mono-output predictive performance across the thirteen modeled outputs.

Binary outcomes are presented as n/N (%), and continuous outcomes as mean +/- standard deviation in days. For binary endpoints, mono-output performance is reported using accuracy, precision, recall/sensitivity, and F1-score after thresholding model outputs at 0.5. The column "Selected predictors retained by mono-output feature selection" lists the variables entering each mono-output model after feature selection. These variables should not be interpreted as SHAP values, permutation feature importance, causal determinants, or ranked outcome-specific predictor effects. Classification metrics were not estimated for ARDS and postoperative in-hospital mortality because each endpoint occurred in only one patient. Estimates for rare endpoints should be interpreted cautiously

Outcome	Outcome type	Prevalence / distribution	Selected predictors retained by mono-output feature selection	Accuracy	Precision	Recall/sensitivity	F1-score	Notes
Atrial fibrillation	Binary	146/513 (28.46%)	n = 5: NYHA class; BMI; hypertension; CKD; COPD	0.602	0.730	0.708	0.719	
Myocardial infarction	Binary	10/513 (1.95%)	n = 1: NYHA class	0.990	0.990	1.000	0.995	Rare endpoint; interpret with caution
Stroke	Binary	6/513 (1.17%)	n = 1: preoperative RBC units	1.000	1.000	1.000	1.000	Rare endpoint; interpret with caution
ARDS	Binary	1/513 (0.19%)	-	-	-	-	-	Not estimated; only one event
Infection	Binary	39/513 (7.60%)	n = 1: age	0.928	0.932	0.996	0.963	
Acute kidney injury	Binary	84/513 (16.37%)	n = 1: BMI	0.866	0.923	0.933	0.928	
Postoperative bleeding	Binary	27/513 (5.26%)	n = 9: preoperative hemoglobin; preoperative RBC units; ASA class; BMI; hypertension; diabetes mellitus; CKD; dyslipidemia; COPD	0.944	0.970	0.971	0.970	
Postoperative in-hospital mortality	Binary	1/513 (0.19%)	-	-	-	-	-	Not estimated; only one event
1-year mortality	Binary	22/513 (4.29%)	n = 1: diabetes mellitus	0.981	0.981	1.000	0.990	Rare endpoint; interpret with caution
1-year reintervention	Binary	27/513 (5.26%)	n = 10: preoperative hemoglobin; preoperative RBC units; age; NYHA class; ASA class; BMI; hypertension; diabetes mellitus; dyslipidemia; COPD	0.944	0.966	0.976	0.971	
1-year hospital readmission	Binary	56/513 (10.92%)	n = 1: diabetes mellitus	0.951	0.951	1.000	0.975	
ICU length of stay	Continuous	Mean +/- SD: 2.71 +/- 3.35 days	Not applicable; continuous outcome	-	-	-	-	Classification metrics not applicable to continuous outcome
Ward length of stay	Continuous	Mean +/- SD: 3.99 +/- 3.17 days	Not applicable; continuous outcome	-	-	-	-	Classification metrics not applicable to continuous outcome

Note. AF, atrial fibrillation; AKI, acute kidney injury; ARDS, acute respiratory distress syndrome; ASA, American Society of Anesthesiologists; BMI, body mass index; CKD, chronic kidney disease; COPD, chronic obstructive pulmonary disease; ICU, intensive care unit; NYHA, New York Heart Association; RBC, red blood cell. Preoperative RBC units refer to units administered before the index operation and do not include intraoperative or postoperative transfusion.
